# Sexual violence and unmet need for contraception among married and cohabiting women in sub-Saharan Africa: Evidence from demographic and health surveys

**DOI:** 10.1371/journal.pone.0240556

**Published:** 2020-11-03

**Authors:** Bright Opoku Ahinkorah, Edward Kwabena Ameyaw, Abdul-Aziz Seidu, Ebenezer Agbaglo, Eugene Budu, Felix Mensah, Collins Adu, Sanni Yaya

**Affiliations:** 1 School of Public Health, Faculty of Health, University of Technology Sydney, Ultimo, Australia; 2 Department of Population and Health, University of Cape Coast, Cape Coast, Ghana; 3 College of Public Health, Medical and Veterinary Sciences, James Cook University, Townsville, Queensland, Australia; 4 Department of English, University of Cape Coast, Cape Coast, Ghana; 5 Fr. Thomas Alan Rooney Memorial Hospital, Asankrangwa, Western Region, Ghana; 6 Department of Health Promotion, and Disability Studies, Kwame Nkrumah University of Science and Technology, Kumasi, Ghana; 7 The George Institute for Global Health, The University of Oxford, Oxford, United Kingdom; 8 School of International Development and Global Studies, University of Ottawa, Ottawa, Canada; University of Salamanca, SPAIN

## Abstract

**Introduction:**

Contraception plays a significant role in fertility regulation and determines the reproductive health rights of women. Studies in other parts of the world have found that sexual violence has negative effects on unmet need for contraception. There has not been any study on the association between these two phenomena in sub-Saharan Africa using current nationally-representative survey data. We investigated the association between sexual violence and unmet need for contraception among married and cohabiting women in sub-Saharan Africa.

**Materials and methods:**

This study was based on secondary datasets from 26 sub-Saharan African countries’ Demographic and Health Surveys conducted between 2010 and 2018. A sample of 101,968 women in sexual unions (married and cohabiting) with complete information on sexual violence and all the variables of interest were included in the analyses. Both bivariate and multilevel logistic regression analyses were carried out to examine the association between sexual violence and unmet need for contraception. Other individual and contextual level socio-economic and demographic variables were considered as covariates. Crude odds ratios [cOR] and adjusted odds ratios [aOR] with their corresponding 95% confidence intervals [CI], signifying precision, were presented. Level of statistical significance was declared at p<0.05.

**Results:**

The overall prevalence of sexual violence and unmet need for contraception in the 26 sub-Saharan African countries were 8.7% and 28.7% respectively. Experience of sexual violence within the last 12 months resulted in 10% increase in unmet need for contraception [OR = 1.10, CI = 1.03–1.14] and 5% increase in unmet need for contraception after controlling for individual and contextual level factors [aOR = 1.05, CI = 1.01–1.11]. With the individual level factors, women with 4 or more births [aOR = 4.85, CI = 4.41–5.33], those cohabiting [aOR = 1.43, CI = 1.37–1.47], those in female headed households [aOR = 1.22, CI = 1.18–1.27] and those who watched television at least once a week [aOR = 1.12, CI = 1.07–1.16] had higher odds of unmet need for family planning. However, those aged 30–34 [aOR = 0.56, CI = 0.52–0.61], those with secondary/higher level of education [AOR = 0.80, CI = 0.77–0.84], those who read newspaper less than once a week [aOR = 0.75, CI = 0.71–0.79] and those who listened to radio at least once a week [aOR = 0.94, CI = 0.90–0.97] had lower odds of unmet need for contraception. In terms of the contextual factors, women in rural areas [aOR = 0.87, CI = 0.84–0.91] and those in the richest wealth quintile households [aOR = 0.80, CI = 0.75–0.85] had lower odds of unmet need for contraception.

**Conclusion:**

Our study has shown an association between sexual violence and unmet need for contraception in sub-Saharan Africa. Experiencing sexual violence increases the likelihood of unmet need for contraception in sub-Saharan Africa. It is also worthy to note that having four or more children, cohabiting with a male partner, and living in female-headed households are some of the key variables associated with unmet need for contraception in sub-Saharan Africa. Our study recommends that, successful contraceptive initiatives should focus on reducing sexual violence, while taking into consideration other significant factors that increase unmet need for contraception. Meanwhile, in doing this, contextual factors ought to be prioritised.

## Introduction

Contraception plays a significant role in fertility regulation and determines the reproductive health rights of women [1]. However, unmet need for contraception acts as a significant barrier to these reproductive health rights [2]. Women with unmet need for contraception refer to those who do not use contraceptive methods despite their desire to space or limit births [3, 4]. Currently, about 214 million women of reproductive age across the world have an unmet need for contraception [5]. Globally, this leads to the occurrence of over 85 million mistimed or unwanted pregnancies each year, ultimately resulting in high rates of unsafe induced abortions, maternal morbidity, and mortality [6]. Legal and safe induced abortions do not pose any substantial adverse implication to maternal health; however, unsafe abortions have enormous consequences for maternal health. Regrettably, over 75% of induced abortions in sub-Saharan Africa (SSA) are unsafe [7]. In low- and middle-income countries, unmet need among married women ranges from 8% in Colombia to 38% in Sao Tome and Principe, with countries such as Haiti, Ghana, and Uganda also recording high prevalence [8].

A number of social, cultural, and economic factors influence women’s decisions regarding contraception [9]. Among the socio-cultural factors, sexual violence has been found to have negative effect on women’s reproductive health issues, including unmet need for contraception [10]. Over the years, sexual violence, being a critical public health concern, has been on the rise and is defined as any sexual act, attempt to obtain a sexual act, or other act directed against a person’s sexuality using coercion, by any person regardless of their relationship to the victim, in any setting [11]. Examples include rape by a husband, boyfriend, stranger, or acquaintance; unwanted sexual advances at school, work, and sexual abuse of mentally or physically disabled people [10]. Much of sexual violence over the world is perpetrated against women in intimate relationships, with a lifetime prevalence among fecund women ranging from 6% in Japan to 59% in Ethiopia [12]. In SSA, the overall prevalence (36%) goes beyond the global average of 30% [13].

A number of studies have investigated whether experiencing intimate partner violence (IPV), which includes sexual violence, is associated with contraceptive use, but results are mixed. Some cross-sectional studies from Europe, India, and Jordan report that women who experience IPV report lower contraceptive use as opposed to women who do not report contraceptive use [14–17] while other studies have come out with contrary findings [18–20]. A recent study in SSA also found no significant association between IPV and contraceptive use in a number of SSA countries where associations were previously reported [21]. Most IPV studies followed quantitative cross-sectional designs and were unable to account for the pathways by which sexual violence causes unmet need. Yet, among the conjectured pathways include the fact that IPV culminates in truncated self-efficacy, thereby reducing chances of contraceptive use [22, 23]. Further, IPV disempowers victims and limits women’s urge for taking absolute control of their reproductive wellbeing. It is also documented that women who experience IPV are less motivated to access or utilize contraceptives [24]. Using demographic and health survey data, Tenkorang [25], Silverman et al [26], and Tsai et al. [27] have examined the associations between IPV and sexual and reproductive health outcomes, including family planning use in Ghana, Niger, and the Philippines, respectively. While Tenkorang [25] found significant associations between IPV and sexual and reproductive health outcomes (unwanted pregnancy and a pregnancy loss) and Silverman et al. [26] found significant associations between IPV and non-use of family planning, the study by Tsai et al. [27], concluded that there was no association between IPV and non-use of family planning.

In the midst of these inconsistent findings, it is not known whether or not there is an association between sexual violence and unmet need for contraception. The present study investigates the association between sexual violence and unmet need for contraception among women of reproductive age in SSA, using the recent Demographic and Health Surveys (DHS) conducted between 2010 and 2018 in 26 SSA countries. This is important, given the prevalence of both sexual violence and unmet need for contraception in the sub-region.

## Materials and methods

### Data source

This study is based on analyses of secondary datasets from 26 SSA DHS conducted between 2010 and 2018. These countries were selected based on the fact that they have current DHS data, have the DHS domestic violence modules in their datasets, and have all the variables considered relevant for this study. DHS collects nationally representative data on demographic, environmental, socioeconomic, nutritional, and health indicators from about 90 low- and middle-income countries. Similar datasets are usually collected across the countries as part of the DHS program usually every five years; hence, they can easily be pooled and used suitably for analysis across multiple countries [27].

The survey usually employs a multi-stage sampling approach across all countries, characterised by three-stage stratified cluster design with household serving as the primary sampling unit. Details of the DHS methodology and data collection procedure have been published elsewhere [28, 29]. Detailed information on the year of survey and sample size for each country has been provided in Table 1. As shown in Table 1, out of the 390,213 women in the 26 countries, 134,505, which represented 34.5%, had complete information on sexual violence in the last 12 months. This corresponds to missing cases of 65.5% on sexual violence, attributed to women who were excluded from the study because they were not eligible to answer questions on sexual violence. These women include those who were either single, divorced, widowed, or separated within the last 12 months prior to the survey. In the present study, a weighted sample of 101,968 women in sexual unions (married and cohabiting) with complete information on sexual violence and all the variables of interest constituted our sample. This sample represented 75.8% of the women with complete information on sexual violence and 24.2% of missing cases. The reason for the sample was that women who had never had sex and those who were infecund/menopausal were further excluded from the main outcome variable (unmet need for contraception) [30–32].

**Table 1:** Detailed description of the study sample.

Survey country	Survey year	Sample^a^	Sample^b^	Sample^c^
Angola	2015–16	14379	7669	6921
Burkina Faso	2017–18	15928	10009	8557
Benin	2018	17087	4488	3566
Burundi	2016–17	17269	7366	5476
DR Congo	2013–14	18827	5680	4160
Cote D’Ivoire	2011–12	10060	5004	3735
Cameroon	2018	13,527	4690	3330
Ethiopia	2016	15683	4720	3805
Gabon	2012	8422	4147	2607
Gambia	2013	10233	3536	2752
Kenya	2014	31079	4511	3395
Comoros	2012	5329	2529	1706
Mali	2018	10519	3356	2892
Malawi	2015–16	24562	5406	4288
Mozambique	2015	7749	3350	1918
Nigeria	2018	41821	8910	7286
Namibia	2013	10018	1448	839
Rwanda	2014–15	13497	1906	1427
Sierra Leone	2013	12352	4315	3163
Chad	2014–15	17719	3801	2838
Togo	2013–14	9480	5370	4002
Tanzania	2015–16	13266	7597	5829
Uganda	2016	18506	7536	5677
South Africa	2016	8514	4003	1671
Zambia	2013–14	13683	7358	5384
Zimbabwe	2015	9955	5800	4743
Total		390,213	134505	101968

^a^Sample size at design

^b^Women with complete information on sexual violence

^c^Women with complete information on all variables of interest

### Definition of variables

#### Dependent variable

The outcome variable for this study is dichotomized as unmet need (yes/no) which was generated from a constructed variable in the DHS. It is the sum of unmet need for spacing and limiting and young women who were married, fecund and/or sexually active have unmet needs if they do not want any more children or want to delay their next birth for at least two years but not using contraception. Multiple reasons may account for the non-use of contraception: the woman herself opposing contraceptive use, other people opposing a woman’s contraceptive use, lack of knowledge, health concerns, lack of access, inconvenient to use, other “fatalistic” reasons (up to God, meaning that the woman feels that pregnancies are determined by fate), and infrequent sex (Moreira et al., 2019) [30]. Pregnant or amenorrheic young women with unwanted or mistimed pregnancies or births were also considered to have unmet need if they were not using contraception at the time they conceived [31, 32].

#### Main explanatory variable

The main explanatory variable of our study was self-reported experience of sexual violence in the last 12 months. There are three standard DHS questions on past year experience of sexual violence: whether the partner ever physically forced the respondent into an unwanted sex; whether the partner ever forced her into other unwanted sexual acts; and whether the respondent has been physically forced to perform sexual acts she didn't want to. For each of these questions, the responses were ‘never’ ‘often’ ‘sometimes’, and ‘yes, but not in the last 12 months’. A dichotomous variable was created to represent whether a respondent had experienced any of these forms of violence in the past 12 months by coding ‘never’ and ‘yes, but not in the last 12 months’ together as ‘No’ and ‘often’ and ‘sometimes’, coded together as ‘Yes’.

#### Covariates

Aside from the main predictor variable (sexual violence), thirteen control variables were considered in our study principally because of their statistically significant relationship with unmet need in previous studies [31, 33–37]. These variables were grouped into individual and contextual level variables. The individual level variables were age, educational level, marital status, working status, frequency of reading newspaper/magazine, frequency of watching television, frequency of listening to radio, parity, decision maker on healthcare, and sex of household head. The contextual level variables were wealth index and place of residence. Age was categorised as 15–19, 20–24, 25–29, 30–34, 35–39, 40–44, and 45–49. Educational level was recoded as “no education,” “primary,” and “secondary/higher.” Marital status was captured as “married” and “cohabiting.” Working status was coded as “working” and “not working”. Frequency of reading newspaper/magazine, listening to radio, and watching television were grouped as “not at all,” “less than once a week,” and “at least once a week.” Parity was categorised as “zero birth”, “one birth” “two births,” “three births” and “four or more births”. Sex of household head was coded as “male” and “female”. Decision maker on healthcare was captured as “respondent alone” and “not alone.” Wealth index was coded as “poorest”, “poorer”, “middle”, “richer” and “richest”. Finally, place of residence was coded as “urban” and “rural”.

### Statistical analyses

In this study, all the analyses were carried out with STATA version 14.0. Pooled data from 101,968 women in 26 SSA countries was used for all the analyses. Apart from the pooling, the following steps were followed to analyse the data. First, we computed the proportion of women with past year experience of sexual violence and unmet need for contraception in SSA and presented them using bar charts. Second, we calculated the prevalence of unmet need for contraception against experience of sexual violence in the last 12 months in addition to all the covariates used in this study with their corresponding confidence intervals. Third, we tested for group-significance of the explanatory variables using chi-square test of independence. This was followed by a bivariate logistic regression analysis to generate crude odds ratios that explain the association between the explanatory variables and unmet need for contraception. Statistical significance for the chi-square test and the bivariate logistic regression were pegged at p<0.05 (Table 2).

**Table 2:** Unmet need for contraception and crude odds ratio by covariates (n = 101,968).

Determinants	Weighted	Weighted	Unmet need	χ2 (p-values)	Bivariate logistic regression		
	N	%	%[95% CI]				
					cOR	Lower	Upper
**Past year experience of sexual violence**				16.6 (<0.001)			
No	90057	91.3	28.4[28.0–28.9]		1	-	-
Yes	8911	8.7	30.0[28.6–31.4]		1.10***	1.05	1.61
**Age**				677.8 (<0.001)			
15–19	7455	7.3	26.4[25.0–27.9]		1	-	-
20–24	19910	19.5	26.1[25.3–27.0]		1.03	0.97	1.10
25–29	23920	23.5	25.7[25.0–26.5]		1.03	0.97	1.10
30–34	20828	20.4	27.0[26.2–27.9]		1.13***	1.06	1.20
35–39	15969	15.7	31.3[30.2–32.5]		1.35***	1.27	1.44
40–44	9692	9.5	36.4[34.9–37.9]		1.62***	1.52	1.74
45–49	4193	4.1	39.0[36.7–41.3]		1.93***	1.77	2.10
**Sex of household head**				90.9 (<0.001)			
Male	86232	84.6	28.0[27.5–28.5]		1	-	-
Female	15736	15.4	31.6[30.5–32.6]		1.19***	1.15	1.24
**Highest educational level**				650.8 (<0.001)			
No education	36886	36.2	32.4[31.7–33.2]		1	-	-
Primary	35245	34.6	29.4[28.6–30.2]		0.87***	0.85	0.90
Secondary /Higher	29837	29.3	22.6[21.8–23.3]		0.64***	0.62	0.66
**Place of residence**				42.1 (<0.001)			
Urban	35788	35.1	27.4[26.5–28.3]		1	-	-
Rural	66180	64.9	29.2[28.6–29.7]		1.10***	1.07	1.13
**Marital status**				347.2 (<0.001)			
Married	78927	77.4	26.9[26.4–27.4]		1	-	-
Cohabiting	23041	22.6	34.5[33.4–35.6]		1.35***	1.31	1.40
**Wealth index**				457.9 (<0.001)			
Poorest	19577	19.2	31.0[30.1–31.9]		1	-	-
Poorer	20643	20.2	30.6[29.7–31.6]		0.99	0.95	1.03
Middle	20585	20.2	29.8[28.9–30.8]		0.95**	0.91	0.99
Richer	20676	20.3	27.9[26.9–29.0]		0.83***	0.80	0.87
Richest	20487	20.1	23.6[22.6–24.6]		0.65***	0.63	0.68
**Frequency of reading newspaper**				492.2 (<0.001)			
Not at all	83305	81.7	30.3[29.7–30.8]		1	-	-
Less than a week	10175	10.0	19.4[18.4–20.4]		0.62***	0.59	0.65
At least once a week	8488	8.3	22.3[20.7–23.9]		0.69***	0.65	0.72
**Frequency of listening to radio**				161.4 (<0.001)			
Not at all	43156	42.3	30.6[30.0–31.3]		1	-	
Less than once a week	19007	18.6	26.5[25.6–27.4]		0.86***	0.82	0.89
At least once a week	39825	39.1	27.3[26.5–28.1]		0.83***	0.81	0.86
**Frequency of watching television**				112.0 (<0.001)			
Not at all	61074	59.9	29.6[29.1–30.2]		1	-	-
Less than once a week	11509	11.3	26.2[26.2–27.3]		0.85***	0.81	0.89
At least once a week	29385	28.8	27.2[26.2–28.2]		0.86***	0.83	0.89
**Parity**				8.2 (<0.001)			
Zero births	5940	5.8	13.6[12.2–15.1]		1	-	-
One birth	15490	15.1	23.7[22.7–24.7]		2.23***	2.05	2.43
Two births	17824	17.5	24.2[23.3–25.1]		2.37***	2.17	2.58
Three births	16098	15.8	26.2[25.3–27.2]		2.56***	2.35	2.79
Four or more births	46716	45.8	34.4[33.7–35.1]		3.81***	3.51	4.13
**Decision maker on healthcare**				0.9 (0.354)			
Respondent alone	18363	18.0	28.6[26.6–29.6]		1	-	-
Not alone	83605	82.0	28.6[28.1–29.1]		1.02	0.98	1.05

*** = p < 0.001

** = p < 0.01and

* = p < 0.05, cOR: crude Odds Ratio, CI Confidence Interval**, 1 = Reference Category**

Finally, we conducted a two-level multilevel logistic regression, where women were nested within clusters and clusters were considered as random effects to cater for the unexplained variability at the contextual level [37, 38]. Five models were generated from the multilevel modelling. The first model was the empty model (model 0), which showed the variation in unmet need for contraception attributed to the distribution of the primary sampling units (PSUs) in the absence of the explanatory variables. Model I had sexual violence and unmet need for contraception only. The purpose was to examine the association between sexual violence and unmet need for contraception, in the absence of the individual and contextual level variables. Model II had only the individual level factors and unmet need for contraception. The purpose for Model II was to look at how the individual level factors only are associated with unmet need for contraception in the absence of the contextual factors. Model III was developed to look at the association between all the contextual level factors and unmet need for contraception, in the absence of the individual level factors. The final model (Model IV) was the complete model that had sexual violence, the individual and contextual level factors and unmet need for contraception. The purpose was to look at the association between sexual violence and unmet need for contraception, after controlling for the individual and contextual level factors. Model comparison was done using the log-likelihood ratio (LLR) and Akaike’s Information Criterion (AIC) tests. Crude odds ratios (cOR) and adjusted odds ratios [aOR] with their corresponding 95% confidence intervals [CI] signifying precision were presented for all the models apart from Model 0 (see Table 2). An odds ratio above 1 indicated higher odds (likelihood) of unmet need for contraception while OR below 1 showed lower odds of unmet need for contraception. The model fitness specification was done with the Hosmer-Lemeshow test while multicollinearity was checked using the variance inflation factor (VIF). The Multicollinearity test showed no evidence of collinearity among the independent variables (Mean VIF = 1.37, Maximum VIF = 1.85, Minimum = 1.02). Sample weight (v005/1,000,000) was applied to correct for over- and under-sampling while the SVY command was used to account for the complex survey design and generalizability of the findings.

### Ethical approval

DHS conforms to the U.S. Department of Health and Human Services regulations for the respect of the rights of human subjects. Additionally, Institutional Review Boards (IRBs) of partner organisations in each of the 26 countries, such as the Ministries of Health gave approval for the survey. During each of the surveys, the women, including those below 16 years, provided either written or verbal consent prior to the data collection. Since the data was publicly accessible, no further ethical approval was required for our study. However, we sought permission from MEASURE DHS website and we were granted the permission to download the dataset on 27^th^ January, 2019. Details about data and ethical standards for DHS are available at http://goo.gl/ny8T6X.

## Results

### Prevalence of unmet need for contraception among married and cohabiting women in sub-Saharan Africa

**Fig 1:**
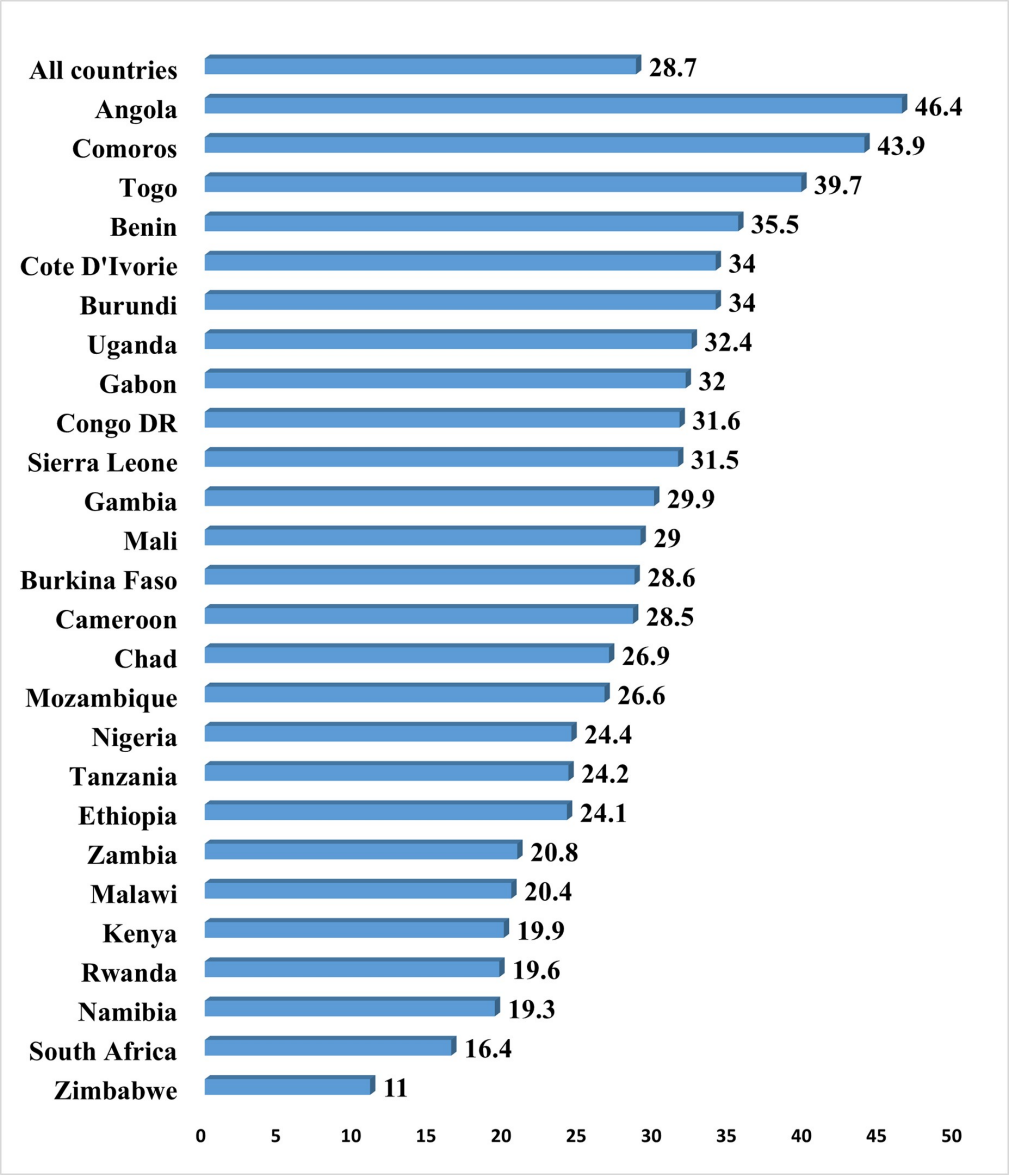
Prevalence of unmet need for contraception among married and cohabiting women in sub-Saharan Africa.

The prevalence of unmet need for contraception among women in the 26 SSA countries included in the study is presented in Fig 1. The overall prevalence of unmet need for contraception was 28.7%, ranging from 11.0% in Zimbabwe to 46.4% in Angola.

### Prevalence of past year experience of sexual violence among married and cohabiting women in sub-Saharan Africa

**Fig 2:**
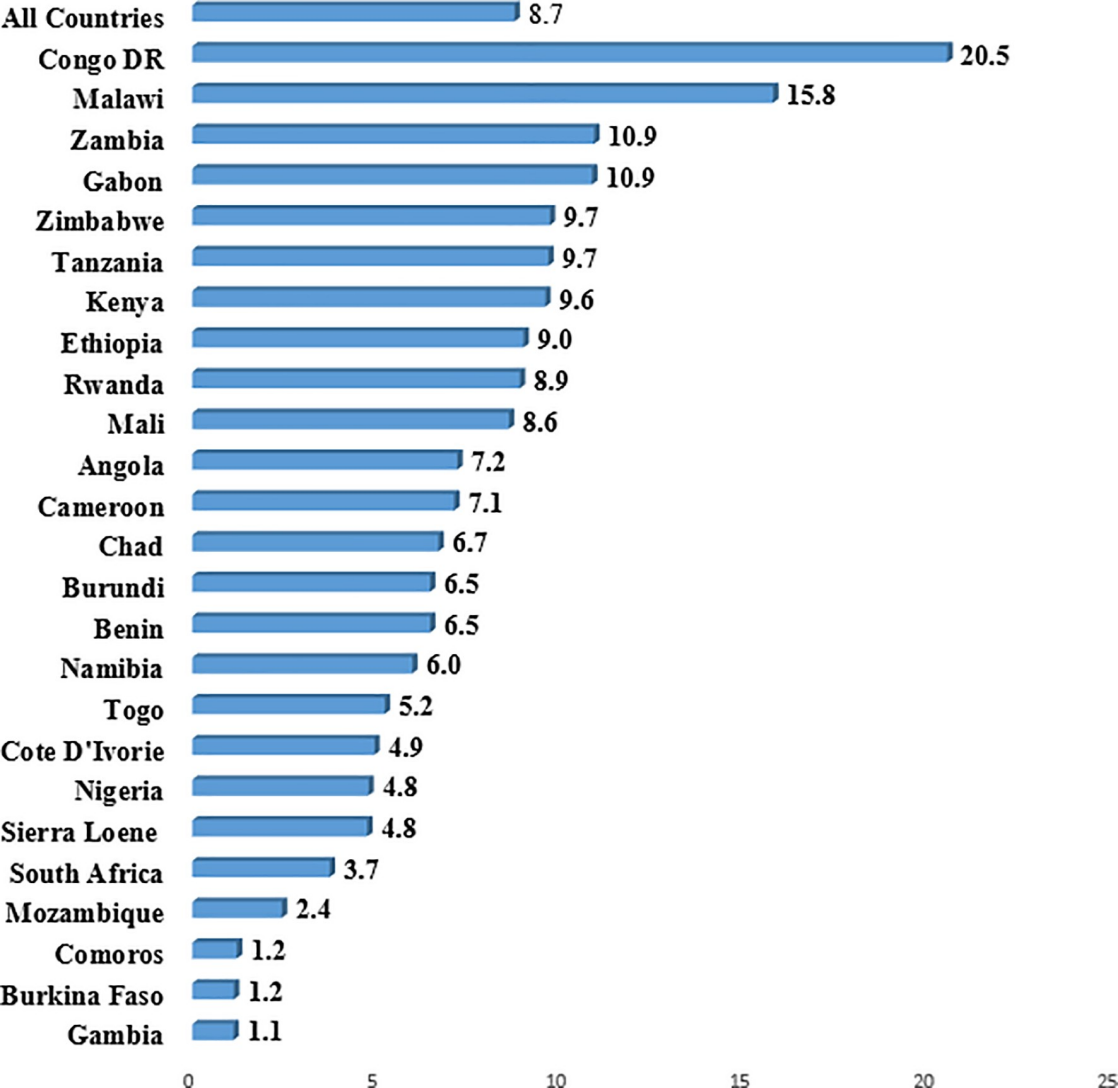
Prevalence of past year experience of sexual violence among married and cohabiting women in sub-Saharan Africa.

The prevalence of past year experience of sexual violence among married and cohabiting in the 26 SSA countries included in the study is presented in Fig 2. The overall prevalence of past year experience of sexual violence was 8.7%, ranging from 1.1% in Gambia to 20.5% in Congo DR.

### Bivariate analysis results on unmet need for contraception across socio-demographic characteristics of women in sub-Saharan Africa

The results showed that 91.3% of the respondents had not experienced sexual violence in the last 12 months and 23.5% of them were aged 25–29. The majority (84.5%) of the respondents were in male headed households. Thirty six percent of the sample had no formal education whereas 29.3% had secondary/higher level of education. About 65% were in rural areas. The majority (77.4%) were married. Approximately 20.3% were in the richer wealth quintile whereas 19.1% were in the poorest wealth quintile. With mass media exposure, 81.7%, 42.3% and 59.9% did not read newspaper, listen to radio nor watch television at all. Approximately 46% had four or more births whereas 5.8% were nulliparous women. The majority (82.0%) did not decide alone on their healthcare (Table 2).

Women aged 45–49 had the highest prevalence (39.0%) of unmet need for contraception compared with those aged 15–19 (26.4%). Women in female-headed households (31.6%), those with no formal education (32.4%), those in rural areas (29.2%), those cohabiting (34.5%), those in the poorest wealth quintile (31.0%), women with parity 4 or more (34.4%) had the highest prevalence of unmet need. With media exposure, it was also found that those who do not listen to radio (30.6%), watch television (29.6%) nor read newspaper at all [30.3%] had the highest prevalence of unmet need for contraception. In addition to the key independent variable (past year experience of sexual violence), all the control variables showed statistically significant association with unmet need for contraception as shown in the results of the chi-square test (Table 2).

The bivariate analysis also showed that women who had experienced sexual violence 12 months to the survey were more likely to have unmet need for contraception [cOR = 1.10, CI = 1.05–1.61]. Women aged 45–49 [cOR = 1.93; CI = 1.77–2.10], those in female-headed households [cOR = 1.19; CI = 1.15–1.24], those in rural areas [cOR = 1.10, CI = 1.07–1.13], those with 4 or more births [cOR = 3.81, CI = 3.51–4.13] and those cohabiting [cOR = 1.35, CI = 1.31–1.40] had the highest odds of unmet need for contraception. Contrariwise, those with secondary or higher level of education [cOR = 0.64, CI = 0.62–0.66], those in the richest wealth quintile [cOR = 0.65, CI = 0.63–0.68], those who read newspaper less than once a week [cOR = 0.62 CI = 0.59–0.65], those who listened to radio at least once a week [cOR = 0.83, CI = 0.81–0.86] and those who watched television less than once a week [cOR = 0.85, CI = 0.81–0.89] had lower odds of unmet need for contraception (Table 2).

### Multilevel logistic regression results on the predictors of unmet need for contraception among women in sub-Saharan Africa

Model 1 of Table 3 shows results of the association between sexual violence and unmet need for contraception, in the absence of the individual and contextual factors. The results indicate that past experience of sexual violence results in 10% increase in unmet need for contraception [OR = 1.10, CI = 1.03–1.14]. After controlling for individual and contextual level factors, past year experience of sexual violence resulted in a 5% increase in the occurrence of unmet need for contraception [aOR = 1.05, CI = 1.01–1.11] (Model 3 of Table 3). This is an indication that sexual violence alone, in the absence of other factors contributes an extra 5% increase to the prevalence of unmet need for contraception. With the individual level factors, women with 4 or more births [aOR = 4.85, CI = 4.41–5.33)], those cohabiting [aOR = 1.43, CI = 1.37–1.47], those in female-headed households [aOR = 1.22, CI = 1.18–1.27] and those who watched television at least once a week [aOR = 1.12, CI = 1.07–1.16] had higher odds of unmet need for family planning. However, those aged 30–34 [aOR = 0.56, CI = 0.52–0.61], those with secondary/higher level of education [aOR = 0.80, CI = 0.77–0.84], those who read newspaper less than once a week [aOR = 0.75, CI = 0.71–0.79] and those who listened to radio at least once a week [aOR = 0.94, CI = 0.90–0.97] had lower odds of unmet need for contraception. In terms of the contextual factors, women in rural areas [aOR = 0.87, CI = 0.84–0.91] and those in the richest wealth quintile [aOR = 0.80, CI = 0.75–0.85] had lower odds of unmet need for contraception (Model 3 of Table 3).

**Table 3:** Multilevel logistic regression results on the predictors of unmet need for contraception among women in sub-Saharan Africa.

Variables	Model 0	Model I	Model II	Model III	Model IV
		OR (95% CI)	aOR (95% CI)	aOR (95% CI)	aOR (95% CI)
**Past year experience of sexual violence**					
No		1	1		1
Yes		1.10*** (1.05–1.16)	1.06* (1.01–1.11)		1.05* (1.01–1.11)
**Age**					
15–19			1		1
20–24			0.75*** (0.72–0.81)		0.76*** (0.71–0.82)
25–29			0.85*** (0.54–0.63)		0.60*** (0.56–0.64)
30–34			0.58*** (0.54–0.63)		0.56*** (0.52–0.61)
35–39			0.61*** (0.56–0.66)		0.63*** (0.58–0.68)
40–44			0.72*** (0.66–0.78)		0.74*** (0.71–0.81)
45–49			0.86*** (0.78–0.95)		0.89** (0.81–0.98)
**Sex of household head**					
Male			1		1
Female			1.23*** (1.19–1.28)		1.22*** (1.18–1.27)
**Highest educational level**					
No education			1		1
Primary			0.89*** (0.86–0.92)		0.89*** (0.86–0.92)
Secondary /Higher			0.79*** (0.76–0.83)		0.80*** (0.77–0.84)
**Marital status**					
Married			1		1
Cohabiting			1.45*** (1.40–1.50)		1.43*** (1.37–1.47)
**Frequency of reading newspaper**					
Not at all			1		1
Less than a week			0.75*** (0.71–0.79)		0.75*** (0.71–0.79)
At least once a week			0.84*** (0.79–0.89)		0.84*** (0.79–0.89)
**Frequency of listening to radio**					
Not at all			1		1
Less than once a week			0.95*(0.91–0.99)		0.96* (0.92–1.00)
At least once a week			0.92*** (0.89–0.95)		0.94*** (0.90–0.97)
**Frequency of watching television**					
Not at all			1		1
Less than once a week			1.01 (0.96–1.06)		1.03 (1.00–1.07)
At least once a week			1.10*** (1.06–1.14)		1.12*** (1.07–1.16)
**Parity**					
Zero births			1		1
One birth			2.47*** (2.26–2.70)		2.46*** (2.25–2.69)
Two births			2.98*** (2.72–3.27)		2.90*** (2.68–3.22)
Three births			3.45*** (3.14–3.79)		3.39*** (3.08–3.72)
Four or more births			4.98*** (4.53–5.47)		4.85*** (4.41–5.33)
**Place of residence**					
Urban				1	1
Rural				0.88*** (0.85–0.91)	0.87*** (0.84–0.91)
**Wealth index**					
Poorest				1	1
Poorer				0.98 (0.94–1.02)	1.02 (0.98–1.07)
Middle				0.93*** (0.89–0.97)	1.00 (0.96–1.05)
Richer				0.79*** (0.75–0.82)	0.92*** (0.88–0.97)
Richest				0.59*** (0.56–0.63)	0.80*** (0.75–0.85)
**Random effect results**					
PSU variance [95% CI]	0.01 (0.01–0.02)	0.01 (0.01–0.02)	0.01 (0.00–0.01)	0.01 (0.01–0.02)	0.01 (0.00–0.02)
ICC	0.003	0.003	0.002	0.003	0.002
LR Test	χ2 = 20.18***	χ2 = 20.18***	χ2 = 15.07***	χ2 = 19.13***	χ2 = 15.27***
Wald χ2	Reference	16.57***	3248.49***	502.65***	3336.40***
Model fitness					
Log-likelihood	-60776.037	-60769.839	-58972.63	-119259.68	-58921.471
AIC	121556.1	121541.7	117991.3	121047.2	117898.9
Number of clusters	1595	1595	1595	1595	1595
N	101,968	101,968	101,968	101,968	101,968

Exponentiated coefficients; 95% confidence intervals in brackets; cOR: crude odds ratios; aOR adjusted odds ratios; CI Confidence Interval

* *p* < 0.05

** *p* < 0.01

*** *p* < 0.001

1 = Reference category PSU = Primary Sampling Unit; ICC = Intra-Class Correlation; LR Test = Likelihood ratio Test; AIC = Akaike’s Information Criterion

## Discussion

Whereas a plethora of evidence attributes unmet need for contraception to sexual violence [32, 39], empirical evidence on this association is unclear in the context of SSA. Nonetheless, SSA accounts for high concentration of unmet need for contraception worldwide [40]. Globally, the highest prevalence of sexual violence occurs in Central (21%) and Southern (17.4%) SSA [13]. With this, we extended previous knowledge by investigating the hypothesis that women who have experienced sexual violence in SSA have greater risk of unmet need for contraception. The overall prevalence of unmet need for contraception was 28.7%. Essentially, women who had experienced sexual violence were more likely to have unmet need for contraception in all our models. Our finding augments previous knowledge from some parts of SSA and diverse global contexts on how sexual violence drives women’s prospects of not meeting their contraceptive needs [14, 16, 32, 39, 41]. We also found an overall prevalence of 8.7% past year experience of sexual violence. In terms of the effects of sexual violence on unmet need for contraception, we found that past year experience of sexual violence resulted in 10% increase in unmet need for contraception. Similar findings on the effect of sexual violence on unmet need for contraception among women have been found in previous studies [25, 26]. Evidence from China further extends the argument by advancing that sexual violence truncates women’s knowledge on sexual and reproductive health, a situation which eventually translates into unmet need for contraception [42].

There are several pathways to explain this finding. Sexual violence may disempower women, thus, compromising their autonomy to negotiate for their preferred method of contraception [43, 44]. This may occur through disapproval by their spouses/husbands or in-laws [42]. On the contrary, an inverse relation between sexual violence and unmet need for contraception has been recounted in literature [19, 20, 45]. An analytical study by the DHS also acknowledged that IPV compromises women’s self-efficacy to control their reproductive life in a manner that aligns with their own fertility aspirations [24]. The same report conjectured that women who experience IPV are less motivated to access or utilize contraceptives [24]. Varied sampling approaches and contextual dynamics may account for these contrary reports. The prevalence of unmet need for contraception found in our study is at par with the rate reported from Zambia (26.6%) [46]. However, it exceeds the 9% of Bostwana [47], 16.1% of Nigeria [41], and the 18% average for the developing world [48]. Our finding and the existing literature suggest the need for SSA governments and contraception stakeholders in SSA to integrate anti-sexual violence campaigns into reproductive health education programs in order to mitigate the prevailing unmet need for contraception.

It is, however, noteworthy that SSA is not homogeneous and substantial contextual, political, and cultural dissimilarities exist [49, 50]. Admittedly, these variations might have accounted for the wide variation in the prevalence for unmet need for contraception such that 11.0% and 46.4% women in Zimbabwe and Angola had unmet need respectively. Beyond IPV, this variation may reflect the level of political will and governments’ investment and commitment to contraceptive services across SSA countries [51].

Women who had four or more births reported high unmet need for contraception. Hitherto, fertility rates across SSA were excessively high, but recent evidence indicates that the sub-region’s fertility rate is 4.7 children per woman [52]. Similar evidence has been reported from Burkina Faso [37]. Rationalising our finding in the context of the nosediving trend of fertility could indicate that women are gradually appreciating the need to reduce childbearing. The desire of women to utilise contraception may signify remarkable impact of fertility reduction advocacies by governments, and other partner organisations such as the International Planned Parenthood Federation (IPPF), which operates in at least 42 countries in SSA [53]. The heightened demand for contraception connotes the possible limited supply or compromised prospects in contraception access. As such, governments and reproductive health advocates ought to transcend beyond advocacies to ensuring easy access and sufficient supply of wide-ranging contraceptives. In light of this evidence, shift from contraception activism to guaranteeing easy access and availability for women with four or higher parity is critical in SSA because demand for contraception is high among women who have high parity [54].

Relative to married women, those cohabiting had unmet need for contraception. Women of female-headed households also had high odds of reporting unmet need for contraception. Considering that out of wedlock childbearing is ostracized in SSA for social, religious and cultural principles [55], this finding is anticipated. The observation that women from female-headed households have high odds of unmet need for contraception concurs with some previous studies from Ghana [35]. This finding could imply that the female household heads are older women (e.g. mother in laws or mothers) and do not approve of contraception because they want their sons to bear many children [56]. Further it is possible these old women did not use any contraception and may neither approve nor realise the need for women in their households to use them either, as commonly observed in the case of female genital mutilation [57]. However, it is not clear in these studies how household head is associated with unmet need for contraception.

Those with secondary or higher level of education had lower odds of unmet need for contraception compared to those without formal education. The finding portrays that educated women, highly educated ones in particular, are well informed and knowledgeable about the possible avenues to satisfy their contraceptive needs [31]. Fagbamigbe et al. [41] similarly noted that women without formal education have high tendency of unmet need for contraception. Our results substantiate the earlier reports from SSA [58], Europe [59] and India [60] and connotes the urgency to prioritize uneducated women in contraceptive initiatives.

We observed that women in the richest wealth quintile and those employed had lower odds of unmet need for contraception. Rich women generally have the financial capacity to purchase any contraception of their choice [35]. This may not be the circumstance of an indigent woman who may be wondering about how to afford three square meals [31]. Asif and Pervaiz [31] explain that rich women have enhanced access to all types of contraceptives compared to the poor. In the same vein, women who are employed may be empowered relative to the unemployed and as a result be better positioned to utilise contraceptive whenever they desire [31]. Those in rural areas had lower odds of unmet need for contraception. Low odds were observed among women aged 30–34 as well. Whilst some prior studies have reported and advanced reasons for high unmet need for contraception among rural women [41, 61], our finding is plausible since some previous interventions have prioritised rural residents [61]. Although health facilities relatively abound in urban locations, women in urban locations may be very busy with tight work demands and therefore have limited time for accessing contraception compared to the rural residents. With respect to age, our finding does not deviate from the account of Wulifan et al. [62] from Ghana. However, it is possible that women in their 30s are still growing their families and have less need for contraception.

Those who read newspaper less than once a week or listened to radio once a week had lower odds of unmet need for contraception. Reading newspaper less than once a week and engagement with the radio once a week generally portray lower engagement with the media. Previous evidence from SSA revealed that high exposure to the media, particularly radio, enhances exposure to family planning messages and reduces the dangers of unmet need for contraception [63–65]. Media has the potency of eroding and demystifying cultural tenets and boosts acceptability and utilisation of contraception [66, 67]. Women’s exposure to contraceptive advocacy messages via media will, therefore, enhance prospects for increasing awareness and knowledge of contraception to attain and sustain women’s interest in contraception usage.

Compared to women in Zimbabwe, women in all the other twenty-five SSA countries had higher odds of unmet need for contraception, with women in Angola having the highest odds. Undeniably, Zimbabwe has witnessed an appreciable increase in contraceptive use over the past decade. For instance, since 2010, there has been a rise from 59% to 67% in the proportion of women aged 15–49 who use contraception in Zimbabwe [68]. This occurrence may account for the less odds of unmet need for contraception relative to other SSA countries. The progress in Zimbabwe did not occur per chance but reflects the concerted effort of the government with Zimbabwe’s Contraceptive Prevalence rate (CPR) standing at 67% which is one of the highest on the continent [68]. The specific initiatives of Zimbabwe can be studied and replicated in other SSA countries, especially Angola, where the highest odds of unmet need occurred.

### Strengths and limitations

The study is supported by comparable nationally-representative data from 26 SSA countries. The representativeness of the data, rigorous methodological approach, and analytical procedure enhance the validity and generalisability of our findings. These notwithstanding, we acknowledge the following limitations. The outcome variable, unmet need for contraception, was self-reported. There is, therefore, the potency for under- or over-reporting as a result of social and cultural ideations. Also, the study followed cross-sectional design and readers ought to interpret our findings in that regard. Again, since data was obtained from countries in different time periods, the likelihood that modernization may affect peoples' awareness of unmet need and sexual violence in recent periods need to be taken into consideration. Moreover, there might be an association of ecological variables at the regional level, in places where there is more prevalence of sexual violence and there might also be less knowledge of contraception due to fear of side effects. Furthermore, this study did not to cater for possible heterogeneity of effects that could have been modelled with a random slope or interaction effects. Finally, the different timing used in the definitions of sexual violence and unmet need may have effect on the interpretation of the results.

## Conclusion

Our study has shown an association between sexual violence and unmet need for contraception in SSA. We found that past experience of sexual violence resulted in 10% increase in unmet need for contraception. It is also worthy to note that having four or more children, cohabiting, living in female-headed households are some of the key predictors of unmet need for contraception in SSA. Based on our analysis, the elimination of sexual violence, a goal in its own, could also lead to a reduction of unmet need for contraception by 10%. SSA countries seeking to enhance contraception coverage and those with relatively high unmet need for contraception like Angola, can under-study Zimbabwe on the measures that have reversed unmet need for contraception to the current level. Meanwhile, in doing this, contextual factors ought to be prioritised.
